# Deciphering the molecular mechanism underlying the effects of epimedium on osteoporosis through system bioinformatic approach

**DOI:** 10.1097/MD.0000000000029844

**Published:** 2022-08-12

**Authors:** Keliang Wu, Linjing Han, Ying Zhao, Qinghua Xiao, Zhen Zhang, Xiaosheng Lin

**Affiliations:** a The Fourth Clinical Medical College of Guangzhou University of Chinese Medicine, Futian District, Shenzhen, Guangdong Province, China; b Guangzhou University of Chinese Medicine, Baiyun District, Guangzhou, Guangdong Province, China; c Integrated Traditional Chinese and Western Medicine Hospital of Shenzhen, Bao’an District, Shenzhen, Guangdong Province, China.

**Keywords:** epimedium, network pharmacology, osteoporosis, traditional Chinese medicine

## Abstract

Epimedium has gained widespread clinical application in Traditional Chinese Medicine, with the functions of promoting bone reproduction, regulating cell cycle and inhibiting osteoclastic activity. However, its precise cellular pharmacological therapeutic mechanism on osteoporosis (OP) remains elusive. This study aims to elucidate the molecular mechanism of epimedium in the treatment of OP based on system bioinformatic approach.

Predicted targets of epimedium were collected from TCMSP, BATMAN-TCM and ETCM databases. Differentially expressed mRNAs of OP patients were obtained from Gene Expression Omnibus database by performing Limma package of *R* software. Epimedium-OP common targets were obtained by Venn diagram package for further analysis. The protein-protein interaction network was constructed using Cytoscape software. Gene Ontology and Kyoto Encyclopedia of Genes and Genomes pathway enrichment analyses were carried out by using clusterProfiler package. Molecular docking analysis was conducted by AutoDock 4.2 software to validate the binding affinity between epimedium and top 3 proteins based on the result of protein-protein interaction.

A total of 241 unique identified epimedium targets were screened from databases, of which 62 overlapped with the targets of OP and were considered potential therapeutic targets. The results of Gene Ontology and Kyoto Encyclopedia of Genes and Genomes enrichment analysis revealed that these targets were positive regulation of cell cycle, cellular response to oxidative stress and positive regulation of cell cycle process as well as cellular senescence, FoxO, PI3K-Akt, and NF-kappa B signaling pathways. Molecular docking showed that epimedium have a good binding activity with key targets.

Our study demonstrated the multitarget and multi-pathway characteristics of epimedium on OP, which elucidates the potential mechanisms of epimedium against OP and provides theoretical basis for further drug development.

## 1. Introduction

Osteoporosis (OP) is a common systemic skeletal disease characterized by reduced bone mass, impaired microarchitecture and susceptible to fracture.^[1]^ Following the accelerated speed of population aging in global, the incidence of OP continues to rise affecting the health and quality of life in the elderly. Currently, the prevention and treatment strategies for OP mainly include bone reproduction promoters, bone resorption inhibitors and bone minerals, such as bisphosphonates, teriparatide, calcitonin, and hormone replacement.^[2]^ However, these drugs may be accompanied by serious adverse effects, including an increasing risk of calculus, jaw necrosis, breast cancer, or atypical femur fracture.^[2–5]^ To avoid the drawbacks of conventional therapies and provide alternative therapeutic agents, uncovering the natural products with potential bone-protecting effects is vitally important.^[6–10]^

Traditional Chinese Medicine (TCM) has been playing an important role in the improvement of life quality and curing disease. In recent years, more and more attentions had been paid to the Chinese herb medicine for the treatment of OP. Epimedium has a longstanding history and has gained widespread clinical application in China, with the functions of promoting bone reproduction, regulating cell cycle, inhibiting osteoclastic activity, and antioxidative stress. In addition, previous studies have reported that epimedium can effectively enhance the activity of osteoblasts, indicating its antiOP biological activities.^[11–13]^ However, its precise cellular pharmacological therapeutic mechanism on OP remains elusive.

Due to the multicomponent, multitarget and multi-pathway characteristics of the TCM in the treatment of diseases, the biological molecular mechanism is relatively complicated. Considerable utilization of manpower and material resources make it difficult to thoroughly investigate the molecular mechanism of TCM through animal and cellular studies.

**Figure 1.**
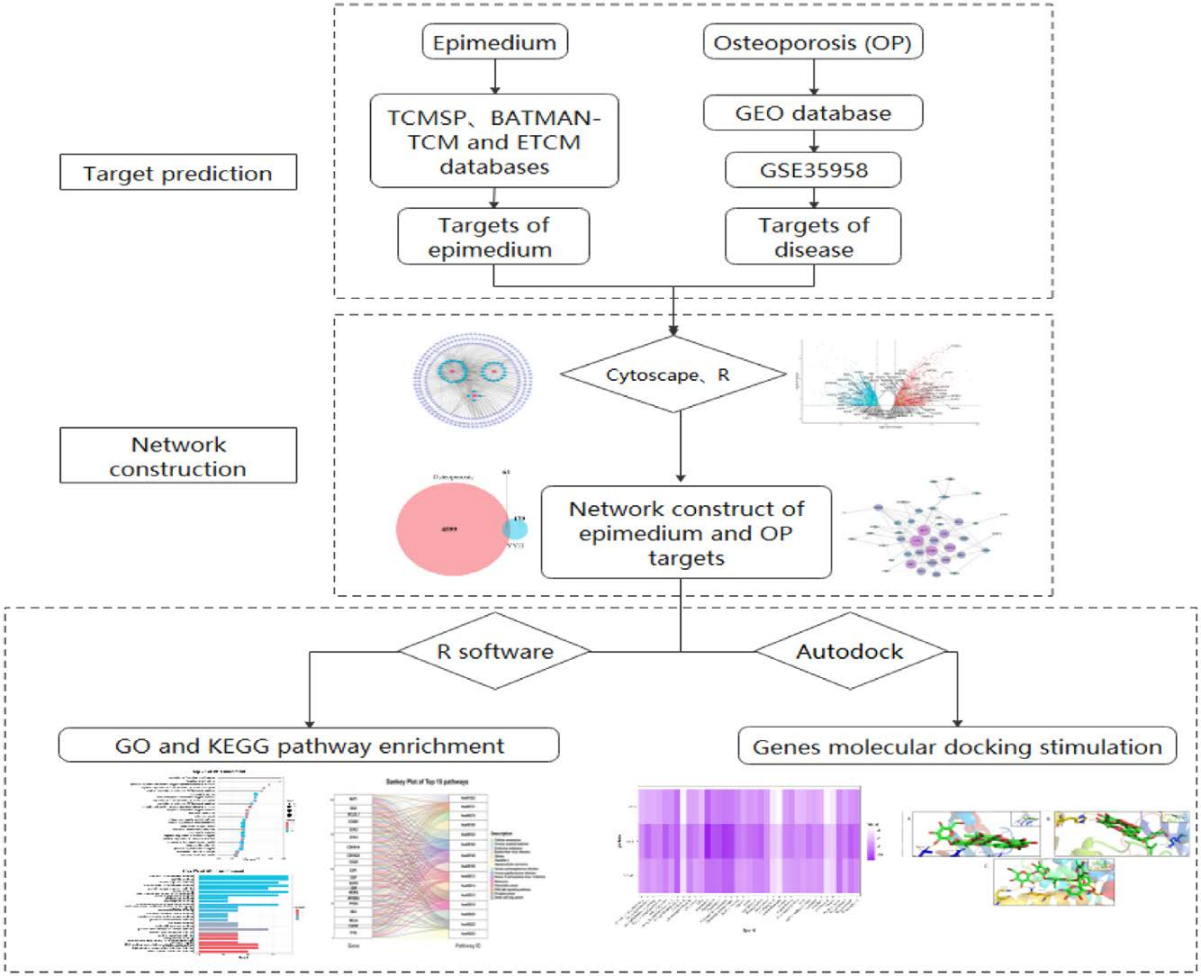
The whole framework based on an integration strategy of network pharmacology.

Network pharmacology is a new subject based on the theory of system biology,^[14]^ which analyzes the network of biological system and selects specific signal nodes to design multitarget drug molecules, including chemoinformatics, bioinformatics, network biology, and pharmacology.^[15,16]^ It emphasizes the multi-channel regulation of signaling pathways, improving the therapeutic effect of drugs and reducing the side effects. Thus, these advantages contribute to improve the success rate of clinical trials of new drugs and save costs for the drug research and development.^[17]^ Therefore, in this study, we adopted the system bioinformatic approach through network pharmacology, molecular docking and microarray data analysis to reveal the pharmacological antiOP mechanism of epimedium. The whole flowchart is shown in Figure 1.

## 2. Materials and Methods

### 2.1. Database building and active compound screening

Epimedium was used as a key word inquire and screen of active ingredients in TCM systems Pharmacology Database and Analysis Platform (TCMSP: http://tcmspw.com/tcmsp.php), Bioinformatics Analysis Tool for Molecular mechanism of Traditional Chinese Medicine database (BATMAN-TCM, http://bionet.ncpsb.org/batman-tcm), and the Encyclopedia of Traditional Chinese Medicine platform (ETCM, http://www.tcmip.cn/ETCM/index.php). TCMSP, one of the world’s largest noncommercial TCM molecular databases, collects 499 herbs from the 2015 Chinese Pharmacopoeia and the compound ingredients of each herb, including 13,144 molecules and 29,384 compounds, in which 2 key absorption, distribution, metabolism, and excretion (ADME) properties including oral bioavailability (OB≥30%) and drug similarity (DL≥0.18) were employed to screen the candidate active compounds in epimedium.^[18]^ In BATMAN-TCM database, effective chemical components were further screened by setting cutoff value to 55. Then, the corresponding targets were entered into UniProt (https://www.uniprot.org/), the most comprehensive and functionally annotated database of protein sequences, and the species was selected as “Homo sapiens.”

### 2.2. The acquisition of differentially expressed genes in OP

The terms “osteoporosis” and “OP” were searched as key words. Gene expression profile of OP named GSE35958 was downloaded from Gene Expression Omnibus database (www.ncbi.nlm.nih.gov/geo), which contained 9 blood samples including 5 OP patients (5 females, mean age 86.2 years) and 4 cases (3 females and 1 male, mean age 81.8 years) as control group. Differential expression analysis between 2 groups was performed to identify differentially expressed mRNAs by setting FDR < 0.05, |logfc| > 0.5 as cutoff threshold. “Limma” package of *R* was used to perform this process.

### 2.3. Network construction for epimedium-OP common targets

By drawing a Venn diagram [VennDiagram of *R* (3.6.2 for Windows)], the intersection of the targets of epimedium and the OP-related targets were obtained as the potential targets for next step analysis. Then, protein-protein interaction (PPI) network was conducted by STRING database (https://string-db.org/), which covered approximately all functional interactions between the expressed proteins.^[19]^ “Homo sapiens” was chosen, and a scoring value >0.4 was selected as the medium confidence basis for protein interactions.^[20]^ Cytoscape (version 3.7.2; https://www.cytoscape.org/) platform was available for visualizing and analyzing the interconnection network, and plug-in “Network Analysis” was used to analyze the topological properties of the network.

### 2.4. Gene ontology and pathway enrichment analysis

The gene ontology (GO) database (http://geneontology.org/), mainly including the biological process (BP) and molecular function (MF) terms, was applied to analyze the potential biological molecular mechanisms. In addition, the Kyoto Encyclopedia of Genes and Genomes (KEGG) database (https://www.kegg.jp/) pathway enrichment analysis was conducted to identify the systemic involvement of pathways. In this research, the GO functional annotation and KEGG pathway enrichment analysis were performed by using clusterProfiler package in *R* software, and the *P* value <0.05 was employed for further analysis.

### 2.5. Molecular docking

Molecular docking analysis was conducted by AutoDock 4.2 software to validate the binding affinity between epimedium and top 3 proteins based on the result of PPI. All 3D structures of the candidate protein targets were obtained from the RCSB Protein Data Bank. Epimedium was prepared by UCSF Chimera.^[21]^ We used the auxiliary program AutoGrid to generate the docking area. In this study, we ignored all the rotations of bound ligands. Finally, the Auto tool was used to calculate the binding energy and inhibition constant between epimedium and its targets. The 3D diagrams of the interactions between epimedium and targets were visualized by PyMOL 1.8.

## 3. Results

### 3.1. Identification targets of epimedium

**Figure 2.**
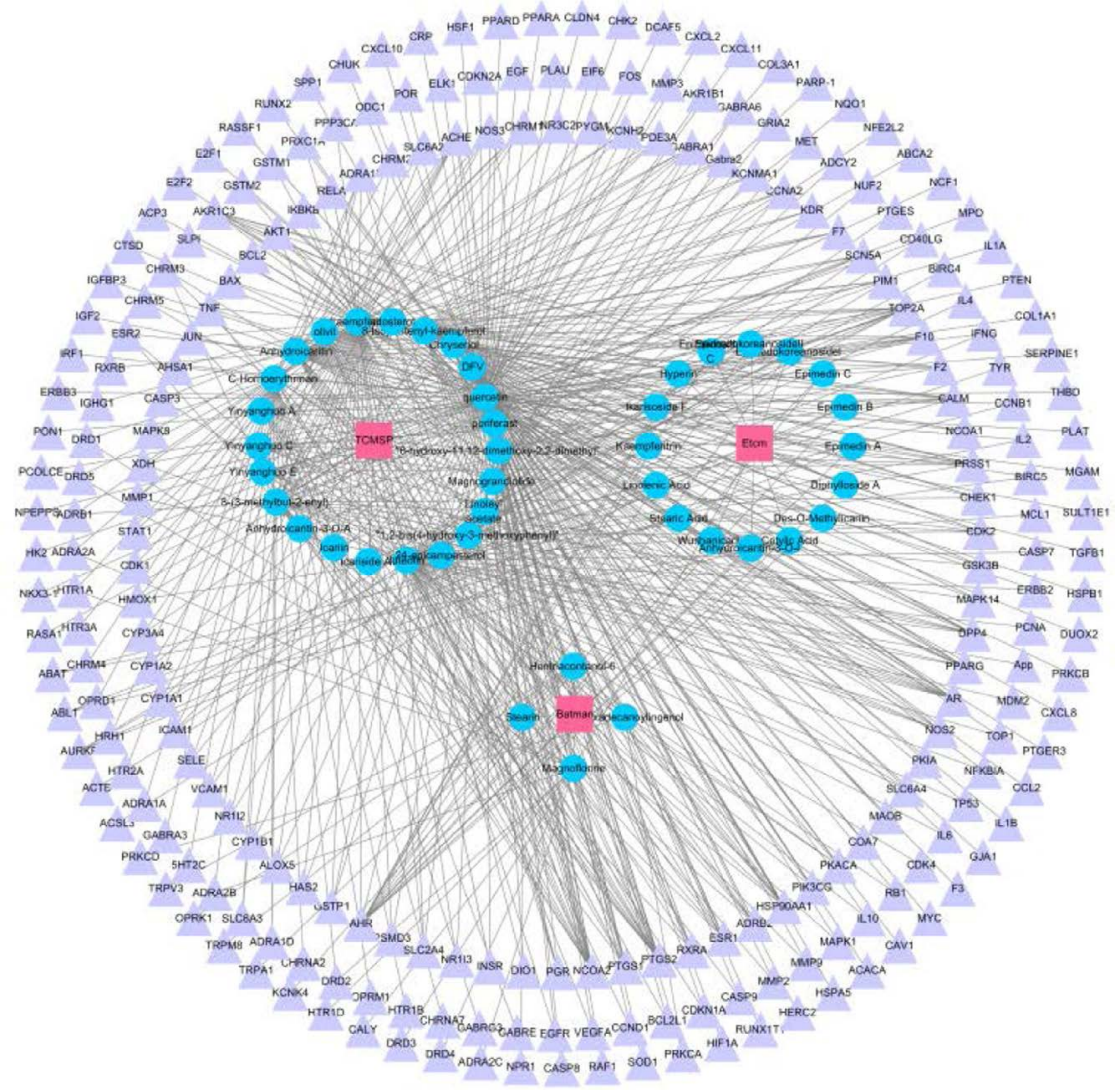
The network of targets for epimedium screened from TCMSP, ECTM, and BATMAN-TCM databases.

The “epimedium” was used as the search term. In TCMSP database, a total of 23 bioactive compounds and 224 predicted targets with OB ≥ 30% and DL ≥ 0.18 were screened out. In BATMAN-TCM database, we obtained 4 bioactive compounds and 18 therapeutic targets with 55 as cutoff threshold. And, in ETCM platform, 17 compounds and 9 targets were retrieved. Finally, a total of 43 kinds of bioactive compounds and 241 unique targets were obtained after taking union set and deleting the duplicate items showed in Figure 2.

### 3.2. Theoretical targets of OP

**Figure 3.**
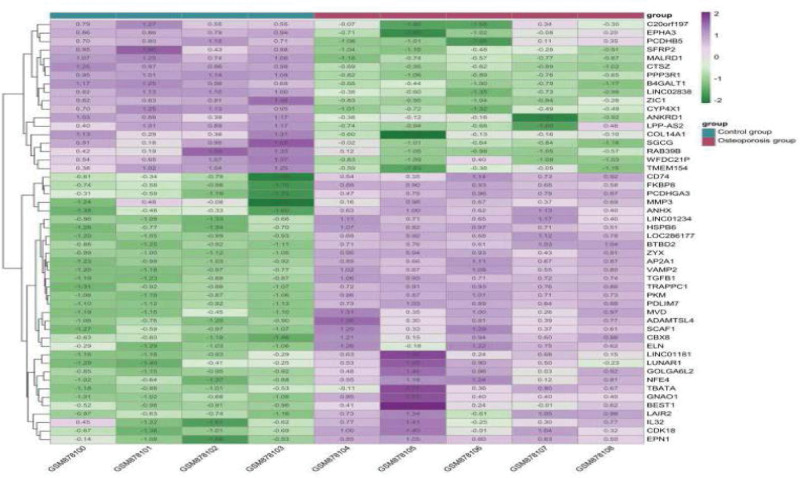
Cluster heat map of differential genes.

Compared with the control group, 4661 differentially expressed genes were detected in blood samples of OP patients, of which 2205 genes were upregulated and 2456 genes were down-regulated. After obtaining the expression matrix of GSE35958 data set, the clustering graph can directly reflect the expression of different genes. Samples of the first 4 groups were control group, and the last 5 groups were OP group. The depth of color represents the level of gene expression, magenta represents the up regulation of gene expression, and green represents the down regulation of gene expression. The first 50 differentially expressed genes were mapped showed in Figure 3.

### 3.3. Common Targets and Compound-Target network

**Figure 4.**
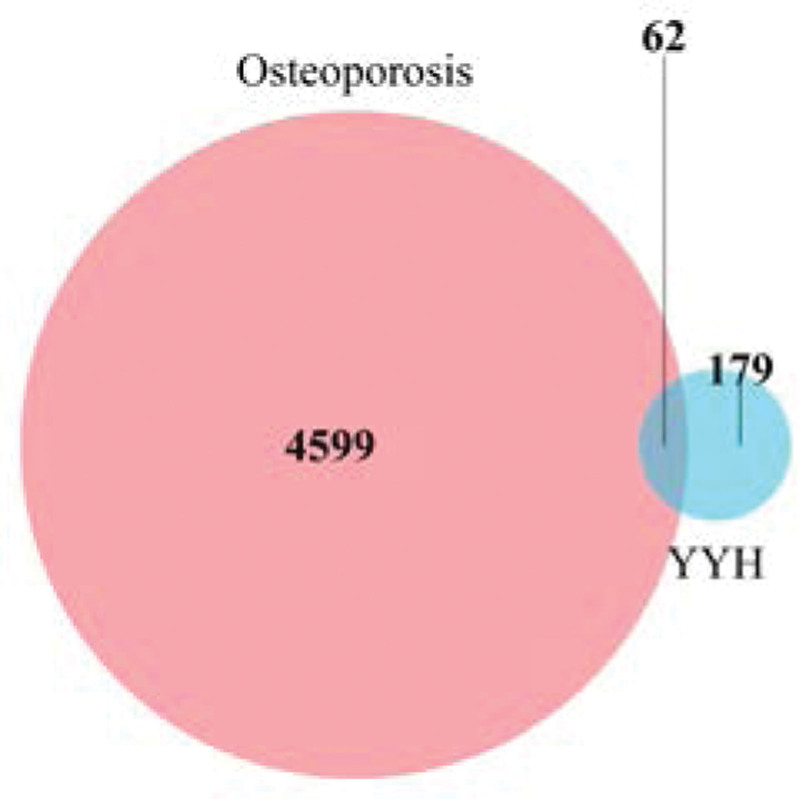
The intersection of epimedium targets and differentially expressed genes in OP patients.

**Figure 5.**
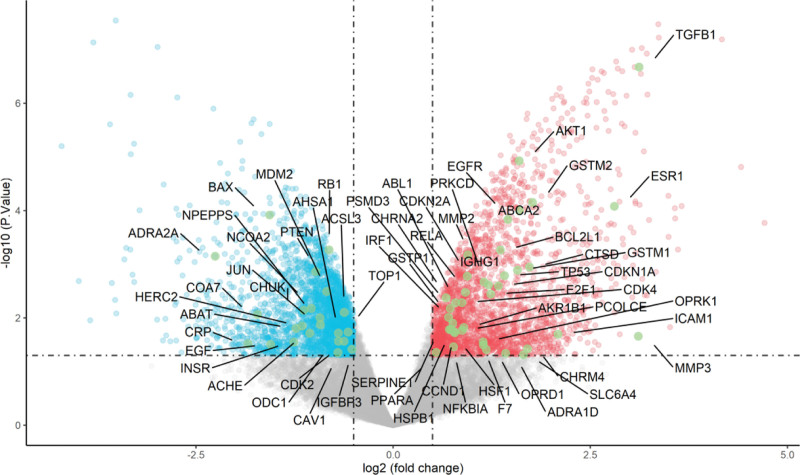
Distribution of 62 therapeutic targets in volcano plot.

**Figure 6.**
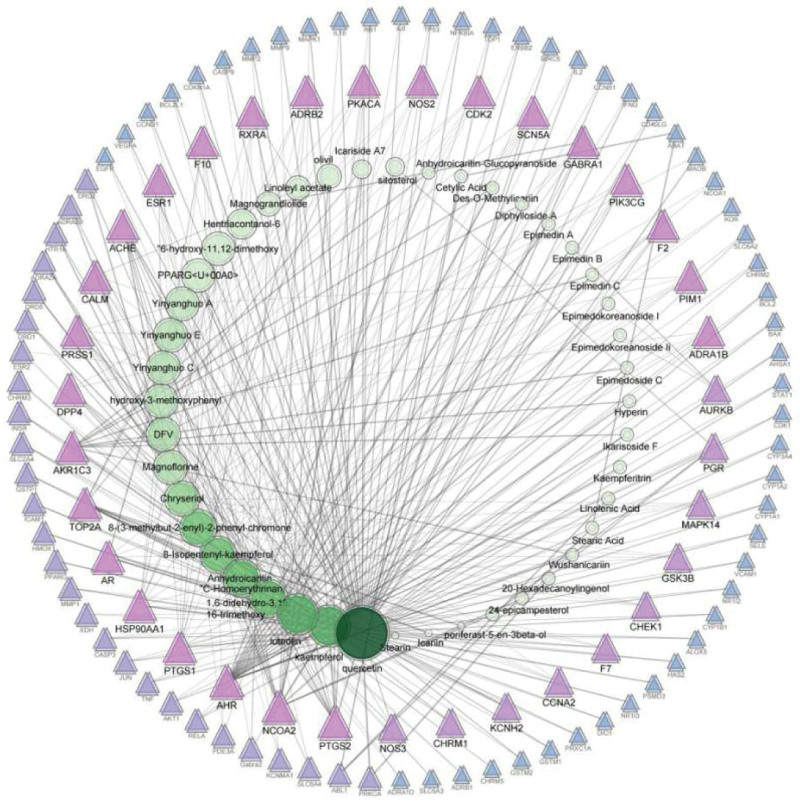
Active compound-target network of potential targets in epimedium. The green nodes represent the potential active compound in epimedium, and the magenta nodes represent the corresponding targets.

The 241 predicted targets of epimedium were intersected with 4661 differentially expressed genes in OP patients, then 62 overlapped targets were obtained as the therapeutic targets through which epimedium exerts an antiOP effect (Fig. 4). Based on the volcano map of original expression matrix, the distribution of 62 therapeutic targets were showed in Figure 5, of which 23 were low expression and 39 were high expression in blood samples. To further directly reflect the interaction relationship between the compounds and their corresponding targets, we constructed a compound-target network by Cytoscape 3.7.2 software (Fig. 6). The topology of the network was analyzed by using the plug-in network analyzer. We found that the network is composed of 157 nodes and 433 edges with deleting degree value ≤ 2 targets. Among them, the top 10 effective components in freedom degree values are as follows: Quercetin, kaempferol, luteolin, C-Homoerythrinan, 1,6-didehydro-3,15,16-trimethoxy-, Anhydroicaritin, 8-Isopentenyl-kaempferol, 8-(3-methylbut-2-enyl)-2-phenyl-chromone, Chryseriol, Magnograndiolide, and DFV. Furthermore, the top 10 therapeutic targets in freedom degree values are as follows: PTGS2, NCOA2, AHR, PTGS1, HSP90AA1, AR, TOP2A, AKR1C3, DPP4, and PRSS1.

### 3.4. Conversion of target proteins into network and analysis

**Figure 7.**
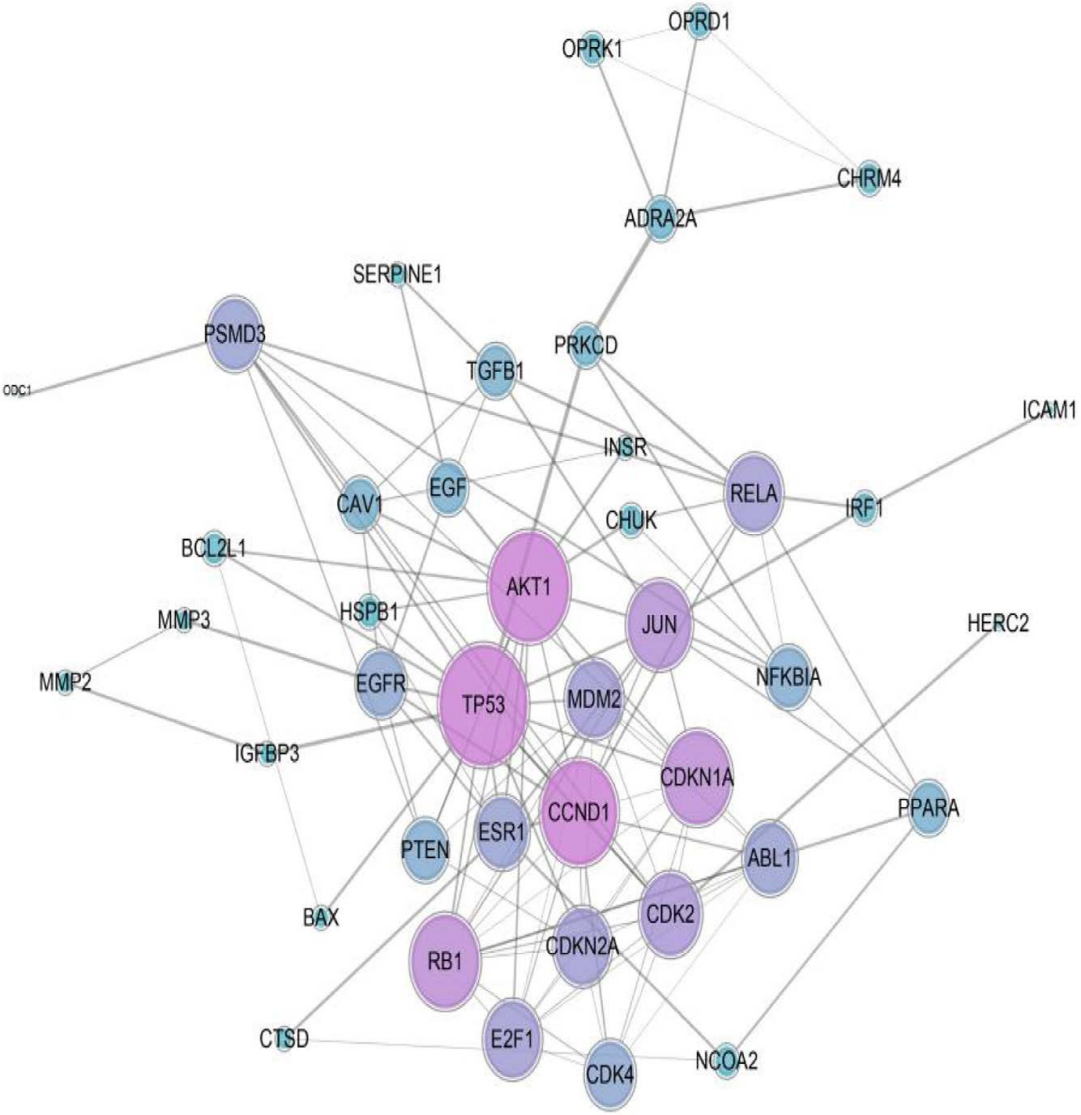
PPI network diagram of epimedium targets.

We then utilized the STRING database in which the minimum required interaction score was set to the high confidence level (0.900) to build the PPI network for the 62 critical potential genes which consists of 42 nodes and 123 edges (Fig. 7). The results were used for further analysis through Cytoscape software to determine Hub proteins. Based on the degree principle, the top 3 targets, namely TP53, AKT1, and CCND1, have higher degree in this process, which explained their importance in the network.

### 3.5. GO enrichment analysis

**Figure 8.**
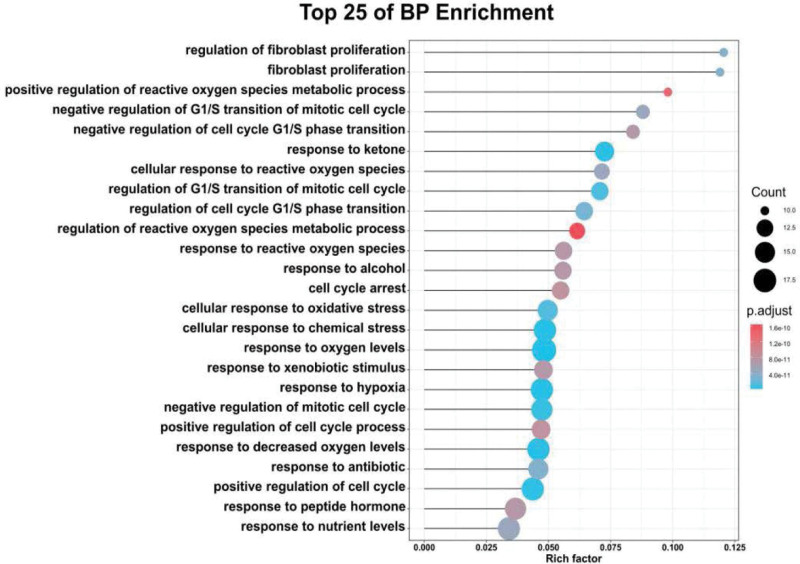
Top 25 Go enrichment analysis for 62 therapeutic targets.

**Figure 9.**
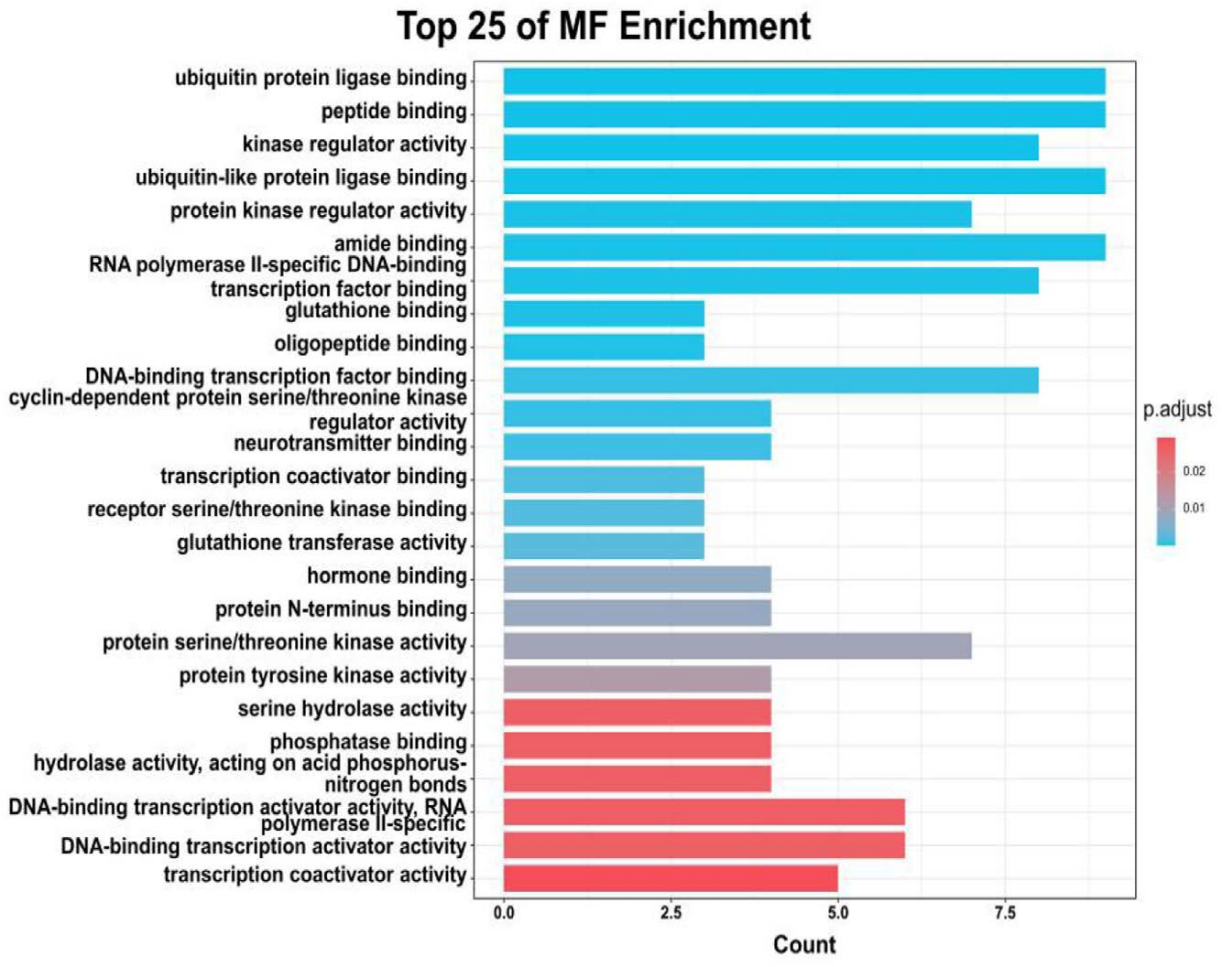
Top 25 Go enrichment analysis for 62 therapeutic targets.

62 common genes were applied to the GO analysis conducted by the clusterProfiler package in *R*. A total of 1340 BP terms were enriched, and the top 25 remarkably enriched BP terms based on *P* value were selected for visualization, including regulation of fibroblast proliferation (GO:0048145), fibroblast proliferation (GO:0048145), positive regulation of reactive oxygen species metabolic process (GO:2000379), cellular response to oxidative stress (GO:0034599), response to decreased oxygen levels (GO:0036293), and positive regulation of cell cycle process (GO:0090068) (Fig. 8). These results indicated that epimedium may play a vital role in the treatment of OP by manipulating these biological processes, which are of great significance to further understand the mechanism of epimedium on OP. A total of 67 MF GO terms were enriched, and the top 25 entries based on *P* value were selected for drawing bubble diagram. These MF terms mainly involved ubiquitin protein ligase binding (GO:0031625), peptide binding (GO:0042277), kinase regulator activity (GO:0019207), and ubiquitin-like protein ligase binding (GO:0044389) (Fig. 9).

### 3.6. KEGG pathway enrichment analysis

**Figure 10.**
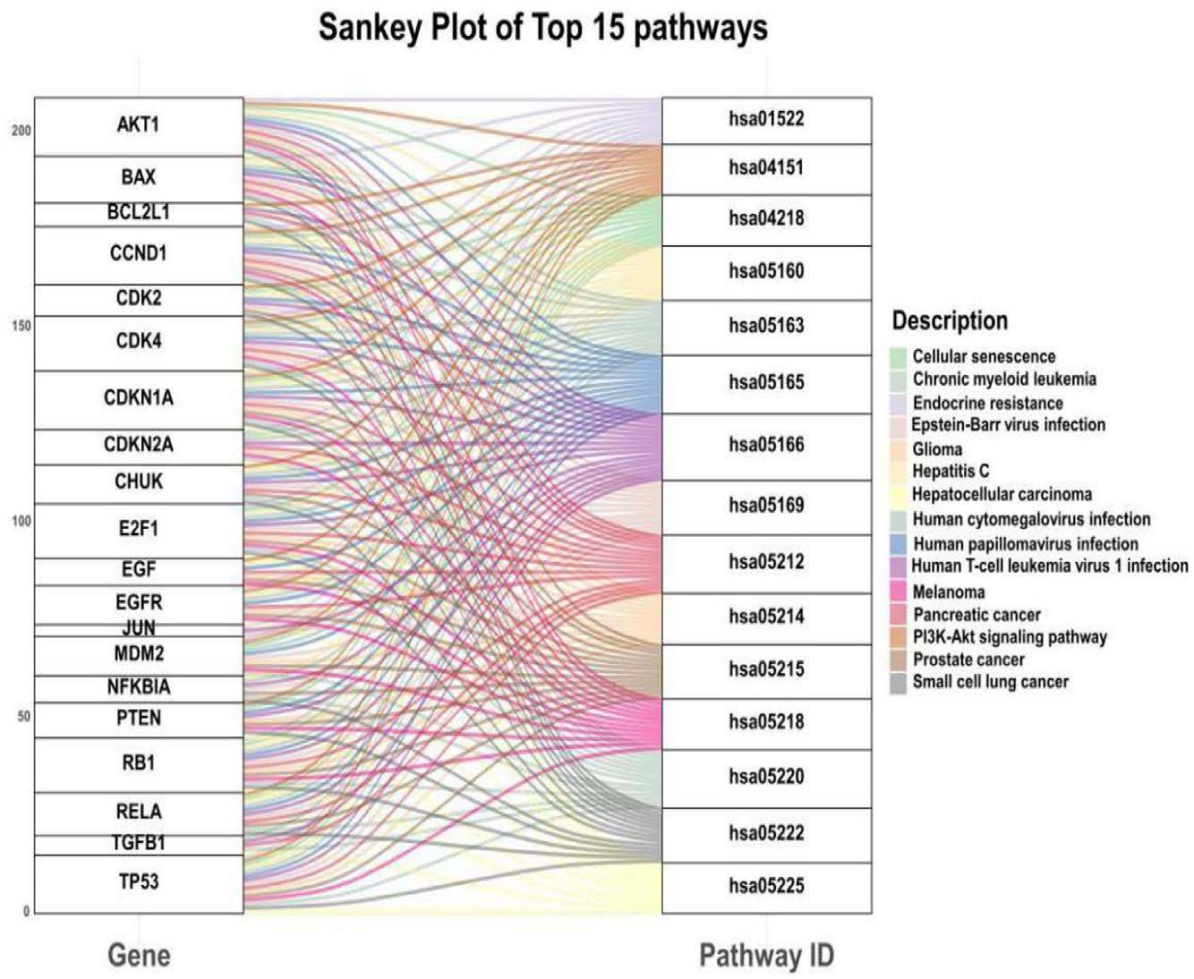
Top 15 pathways of KEGG enrichment analysis.

To further uncover the potential mechanisms of epimedium against OP at the pathway level, we adopted the clusterProfiler package to conduct a KEGG enrichment analysis on 62 targets and screened out top 15 pathways based on the threshold of *P* value. And, the critical gene-pathway network showed that AKT1, CCND1, CDK4, CDKN1A, E2F1, RB1, TP53 etc were the frequently enriched genes and may play key roles in the effects exerted by epimedium (Fig. 10). Many pathways for potential target genes were identified, such as Cellular senescence, PI3K-Akt signaling pathway and mTOR signaling pathway are related to autophagy activity. HIF-1 signaling pathway and FoxO signaling pathway are associated with signal transduction. TNF signaling pathway NF-kappa B signaling pathway and IL-17 signaling pathway are related to the inflammatory. Moreover, toll-like receptor signaling pathway and focal adhesion are closely related to immunological stress. In addition, we found some other pathways such as AGE-RAGE signaling pathway in diabetic complications, Chronic myeloid leukemia, Hepatitis C, Endocrine resistance and Inflammatory bowel disease, which uncovered that epimedium also has a potential application in other diseases. The results showed that epimedium played key roles in antiOP effect mainly by regulating antioxidant stress, immunization, and inflammatory reaction.

### 3.7. Docking stimulation verification

**Figure 11.**
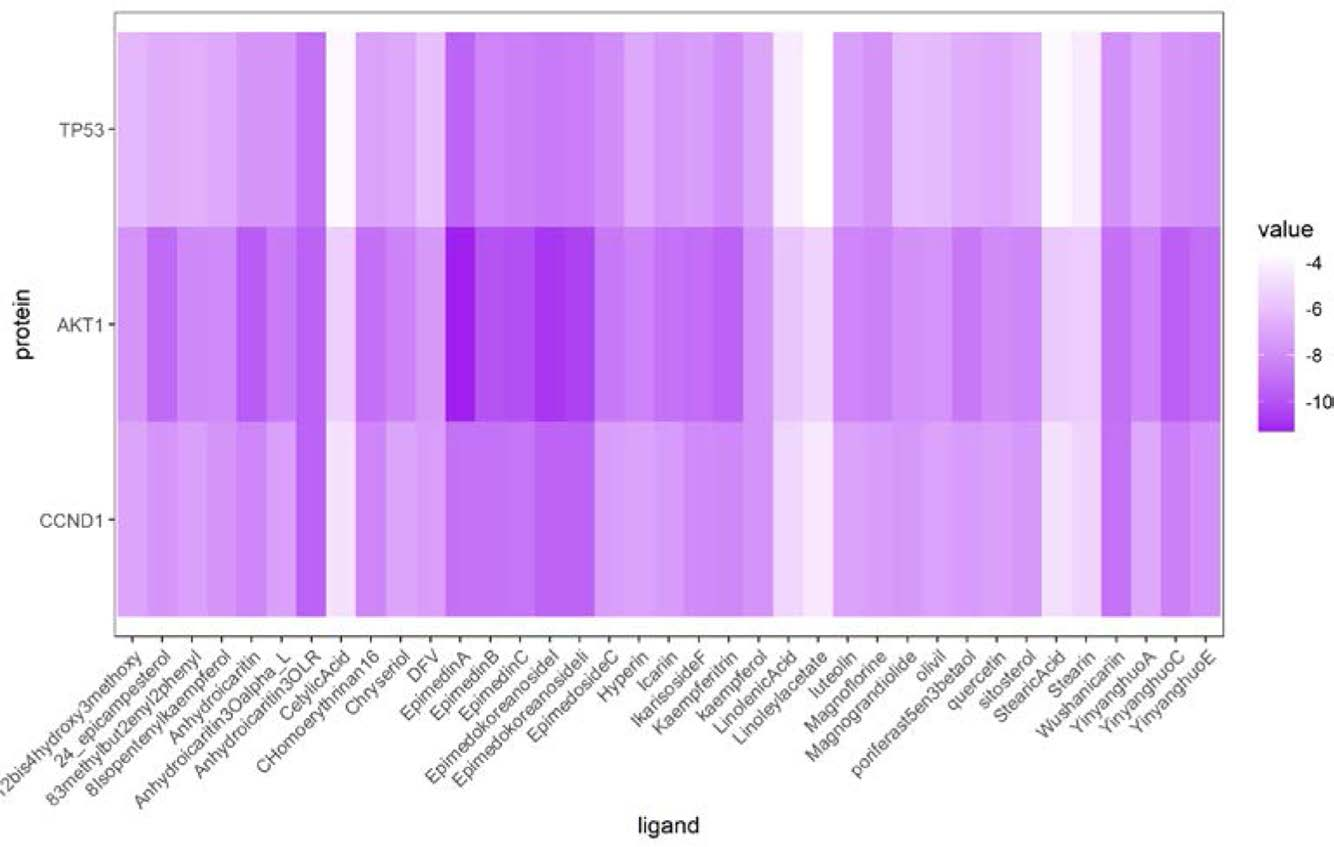
Heat maps of the docking scores of hub genes combining with bioactive compounds in epimedium.

**Figure 12.**
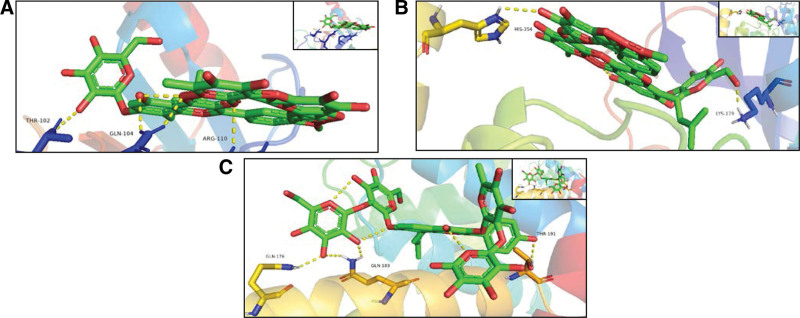
Molecular docking stimulation of bioactive compound-hub gene (A) EpimedinA to TP53 (docking score = −9.4); (B) EpimedinA to AKT1 (docking score = −11.3); (C) Anhydroicaritin3OLR to CCND1 (docking score = −9.5).

In this study, we used the molecular docking stimulation in order to identify the binding ability between components of epimedium and the obtained hub genes. As showed in Figure 11, all the bioactive components demostrated a good binding with these genes. Among these genes, the top 3 hub genes, namely TP53, AKT1, and CCND1, interacted well with the related active compounds (Fig. 12).

## 4. Discussion

OP is characterized by low bone mineral density and higher risk of fracture due to bone fragility. The side effects caused by long-term use of antiOP drugs are gradually garnering public concern.^[2–5]^ TCM have been playing an important role in the prevention and treatment of OP due to its safety and effectiveness. Herb epimedium is one of the most extensively used herbs in antiOP traditional herbal drugs prescriptions, but its molecular mechanism is still not fully understood. Therefore, in the present study, we constructed multi-tiered network to predict drug targets by using a pharmacological drug discovery approach which included identification of the gene-targets and the use of the multiple components-multiple targets-multiple pathways-multiple disease approach. Moreover, the stable molecular docking model of the compound-target showed effective binding in representative compounds and hub OP targets verifying the inner links between epimedium and OP.

Through the screening of active ingredients and analysis of compound-target network, quercetin, kaempferol, and luteolin were identified to be the most important active ingredients of epimedium against OP. Many studies have confirmed that quercetin could promote the proliferation of bone marrow mesenchymal stem cells and exert antiinflammatory effects to inhibit bone loss.^[22]^ Also, it was reported that quercetin could reduce the oxidative damage in MC3T3-E1 osteoblast-like cells induced by peroxide via down-regulating part of the toxic transduction pathway.^[23]^ Masaaki et al^[24]^ have found that quercetin could reduce bone resorption by inhibiting osteoclastogenesis related to its estrogenic activity. Luteolin has been reported to exhibit the antioxidant activity in glucocorticoid-induced osteoporosis by regulating the ERK/Lrp-5/GSK-3β pathway in vivo and in vitro.^[25]^ Notably, these active components synergistically exert antiinflammatory, maintenance of estrogen level and antioxidative stress bone formation, which can be regarded as potential therapeutic strategies on OP.

A total of 241 known targets of epimedium were found by using network pharmacology, indicating its wide range of therapeutic effects and good biological activity. Among all targets, 62 therapeutic targets were obtained as the key targets through which epimedium exerts an antiOP effect. Then, we concluded that many targets can be adjust by multiple compounds, such as PTGS2, NCOA2, AHR, PTGS1, and AKR1C3. This confirmed that epimedium has the biological characteristics of multicomponent and multitarget in treating OP. The PPI network highlighted pivotal targets and interaction degree with others, such as TP53, AKT1, and CCND1. In the entire target network of epimedium against OP, these targets showed a rich interaction with others and were more likely to produce a cascade effect in the treatment of OP.

In order to reveal the mechanism of epimedium against OP, we analyzed the candidate targets by performing GO enrichment via biological processes and molecular function. The main GO biological processes terms related to OP including positive regulation of cell cycle (*P* value = 4.32E-15, ranked in 6^th^), cellular response to oxidative stress (*P* value=3.47E-14, ranked in 9^th^), positive regulation of cell cycle process (*P* value = 5.9E-13, ranked in 22^nd^) and regulation of apoptotic signaling pathway (*P* value = 2.52E-12, ranked in 26^th^). What’s more, the protein serine/threonine kinase activity and transcription co-factor binding were analyzed by molecular function enrichment analysis. These results suggested that antiOP mechanisms mainly involved antioxidative stress, regulation of cell cycle and apoptosis. Studies have revealed that oxidative stress plays an important role in the pathogenesis of OP.^[26,27]^ Oxidative stress inhibits osteoblast differentiation and stimulates osteoclast differentiation in bone marrow, leading to OP and accelerating its progress. More importantly, oxidative stress can accelerate cell senescence and death by activating many signal transduction pathways, including inflammatory transcription pathway nuclear factor (NF-κb), JAK/STAT signaling, mitogen activated protein kinases (MAPKs), and heat shock factor (HSF).^[28]^ Chavan et al^[29]^ have found that the oxidative biological markers in postmenopausal OP women were significantly higher than those in the healthy group. The positive regulation of cell cycle and apoptosis process of epimedium could balance bone reproduction and bone loss, thus delaying the process of OP.

The therapeutic targets of epimedium were enriched in several OP-related pathways, such as cellular senescence (*P* value = 8.52E-14, ranked in 12^nd^), FoxO signaling pathway (*P* value = 1.11E-09, ranked in 24^th^), PI3K-Akt signaling pathway (*P* value = 8.48E-08, ranked in 31^st^), and NF-kappa B signaling pathway (*P* value = 0.00074, ranked in 69^th^). Under the background of global aging, cellular senescence has received considerable attention as a potential target in treating multiple age-related diseases such as osteoporosis. Sundeep et al^[30]^ have found the expression of senescent cell biomarker Cdkn2a increased consistently in mice with aging in bone-derived enriched populations of osteoprogenitors, osteoblasts, and osteocytes. In addition, researchers also found the expression levels of senescence biomarkers p16INK4A and p21CIP1 were statistically significantly increased in iliac crest of older postmenopausal women compared with younger premenopausal women.^[31]^ These data indicated that senescent cells accumulate in old bone in mice and humans, implicating a potential role for these cells in the pathogenesis of OP. Our result showed that epimedium could prevent and treat OP through regulating cellular senescence.

Also, the KEGG enrichment analysis suggested that epimedium exerted antiOP through FoxO signaling pathway, PI3K-Akt signaling pathway and NF-kappa B signaling pathway (*P* value = 0.00074, ranked in 69^th^), which are closely related to OP. FoxOs are evolutionarily conserved transcription factors for their highly conserved DNA-binding domain. FoxOs participate in regulating many cellular processes, including cell survival, proliferation, differentiation, apoptosis, oxidative stress resistance, metabolism, inflammation, and aging.^[32–34]^ Recent studies have revealed that FoxOs regulate bone cell functions and their intercellular signaling. It is reported that H_2_O_2_ accumulation is a critical adaptation for the differentiation and survival of osteoclasts. Bartell et al^[35]^ confirmed that FoxO proteins could inhibit the osteoclast formation and bone resorption through attenuating H_2_O_2_ accumulation. In addition, the PI3K-Akt signaling pathway plays an important role in differentiation of bone cells, especially in osteoblasts.^[36]^ PI3K is a lipid kinase that generates PIP3, which recruits and activates PDK-1 and Akt. Akt is a major signaling molecule activated by PI3K and plays a key role in osteoblast differentiation. Previous study has reported that blocking the PI3K/Akt signaling pathway can heavily inhibit cell proliferation, calcium accumulation and ALP activity in osteoblasts.^[37]^

NF-κB is a key transcription factor in cells, which is involved in antioxidative stress, inflammatory regulation, immune response, cell differentiation, and apoptosis. NF-κB signaling pathway is one of the most important signaling pathways linked to the loss of skeletal muscle mass in normal physiological and pathophysiological conditions.^[38]^ Although muscle atrophy might involve differential activation of multiple cell signaling pathways, recent evidence suggest that NF-κB is one of the most important signaling systems, the activation of which leads to skeletal muscle loss. Zhu et al^[39]^ have found that tanshinone IIA had protective effects against oxidative stress in osteoblastic differentiation in mice with osteoporosis by regulating the NF-κB signaling pathway. Huang et al^[40]^ confirmed that Mettl21c might exert its bone-muscle pleiotropic function via the regulation of the NF-κB signaling pathway, which is critical for bone and muscle homeostasis.

The top 3 hub gene targets, namely TP53, AKT1, and CCND1, enriched in these key pathways were selected for molecular docking studies. These docking results suggested good affinity of epimedium toward these 3 targets and validated above network pharmacology results.

However, there were some unavoidable limitations in our study. The predicted targets of quercetin were collected from 3 main databases. It was insufficient for the investigation of quercetin mechanism. Nevertheless, through a series of network pharmacological methods and including many previous research results, we revealed the main therapeutic targets, which provided general therapeutic targets and references for the treatment of OP. In the future, interrupting candidate genes via Clustered Regularly Interspaced Short Palindromic Repeats or modifying their expression via small, interfering RNA could also help infer MF, providing better annotation and aid their integration into pathways. Finally, although network pharmacology is an efficient and convenient method for predicting therapeutic targets in sophisticated diseases, it is still necessary to verify the rationality of the predicted targets through vivo and vitro experiments.

## 5. Conclusions

Based on bioinformatics and network pharmacology analysis, this study revealed the molecular mechanism underlying the effect of epimedium on OP, which is consistent with previous studies. These results demonstrated the multitarget and multipathway mechanisms of epimedium against OP, which affords the following drug research the promising direction and theoretical basis. However, in vivo and in vitro experiments are further required to verify above therapeutic mechanisms.

## Acknowledgments

This study was supported by the Shenzhen Science and technology innovation Commission (JCYJ20180302144355408, JCYJ20190808100818959). The sponsor has no role in study design; in the collection, analysis and interpretation of data; in the writing of the report; and in the decision to submit the article for publication.

## Author contributions

Data curation: Keliang Wu, Linjing Han, Yingzhao, Qinghua Xiao, Zhen zhang.Funding acquisition: Xiaosheng Lin.Investigation: Keliang Wu, Zhen zhang, Linjing Han.Project administration: Keliang Wu, Yingzhao, Xiaosheng Lin.Writing – original draft: Keliang Wu, Linjing Han.Writing – review & editing: Keliang Wu.

## References

[1] Wang N Xu P Wang X. Integrated pathological cell fishing and network pharmacology approach to investigate main active components of Er-Xian decoction for treating osteoporosis. J Ethnopharmacol. 2019;241:111977. 31136804 10.1016/j.jep.2019.111977

[2] Wong SK Chin KY Ima-Nirwana S. The osteoprotective effects of kaempferol: the evidence from in vivo and in vitro studies. Drug Des Devel Ther. 2019;13:3497–514. 10.2147/DDDT.S227738 PMC6789172 31631974

[3] Che J Liang B Zhang Y. Kaempferol alleviates ox-LDL-induced apoptosis by up-regulation of autophagy via inhibiting PI3K/Akt/mTOR pathway in human endothelial cells. Cardiovasc Pathol. 2017;31:57–62. 28985493 10.1016/j.carpath.2017.08.001

[4] Einhorn TA Bogdan Y Rd TP. Bisphosphonate-associated fractures of the femur: pathophysiology and treatment. J Orthop Trauma. 2014;28:433. 24121986 10.1097/BOT.0000000000000023

[5] Tabatabaei-Malazy O Salari P Khashayar P. New horizons in treatment of osteoporosis. Daru. 2017;25:2. 28173850 10.1186/s40199-017-0167-z PMC5297185

[6] Kuan WS Kok-Yong C Soelaiman I. The effects of tocotrienol on bone peptides in a rat model of osteoporosis induced by metabolic syndrome: the possible communication between bone cells. Int J Environ Res Public Health. 2019;16:3313. 10.3390/ijerph16183313 PMC6765824 31505801

[7] Wong SK Chin K Suhaimi FH. The effects of palm tocotrienol on metabolic syndrome and bone loss in male rats induced by high-carbohydrate high-fat diet. J Funct Foods. 2018;44:246–54.

[8] Wong SK Chin KY Ima-Nirwana S. Berberine and musculoskeletal disorders: the therapeutic potential and underlying molecular mechanisms. Phytomedicine. 2020;73:152892. 30902523 10.1016/j.phymed.2019.152892

[9] Wong SK Chin KY Suhaimi FH. Exploring the potential of tocotrienol from Bixa orellana as a single agent targeting metabolic syndrome and bone loss. Bone. 2018;116:8–21. 29990585 10.1016/j.bone.2018.07.003

[10] Wong SK Mohamad NV Ibrahim N’. The molecular mechanism of vitamin E as a bone-protecting agent: a review on current evidence. Int J Mol Sci. 2019;20:1453. 10.3390/ijms20061453 PMC6471965 30909398

[11] Xi HR Ma HP Yang FF. Total flavonoid extract of Epimedium herb increases the peak bone mass of young rats involving enhanced activation of the AC10/cAMP/PKA/CREB pathway. J Ethnopharmacol. 2018;223:76–87. 29783019 10.1016/j.jep.2018.05.023

[12] He J Zang S Liu N. Epimedium polysaccharides attenuates hematotoxicity by reducing oxidative stress and enhancing immune function in mice model of benzene-induced bone marrow failure. Biomed Pharmacother. 2020;125:109908. 32014688 10.1016/j.biopha.2020.109908

[13] Kim DR Lee JE Shim KJ. Effects of herbal Epimedium on the improvement of bone metabolic disorder through the induction of osteogenic differentiation from bone marrow-derived mesenchymal stem cells. Mol Med Rep. 2017;15:125–30. 27959402 10.3892/mmr.2016.6015 PMC5355742

[14] Eisenhardt S Fleckenstein J. Traditional Chinese medicine valuably augments therapeutic options in the treatment of climacteric syndrome. Arch Gynecol Obstet. 2016;294:193–200. 27040419 10.1007/s00404-016-4078-x

[15] Chen L Cao Y Zhang H. Network pharmacology-based strategy for predicting active ingredients and potential targets of Yangxinshi tablet for treating heart failure. J Ethnopharmacol. 2018;219:359–68. 29366769 10.1016/j.jep.2017.12.011

[16] Zhang J Liang R Wang L. Effects and mechanisms of Danshen-Shanzha herb-pair for atherosclerosis treatment using network pharmacology and experimental pharmacology. J Ethnopharmacol. 2019;229:104–14. 30312741 10.1016/j.jep.2018.10.004

[17] Nie H Deng Y Zheng C. A network pharmacology-based approach to explore the effects of Chaihu Shugan powder on a non-alcoholic fatty liver rat model through nuclear receptors. J Cell Mol Med. 2020;24:5168–84. 32189432 10.1111/jcmm.15166 PMC7205817

[18] Pang HQ Yue SJ Tang YP. Integrated metabolomics and network pharmacology approach to explain possible action mechanisms of Xin-Sheng-Hua granule for treating anemia. Front Pharmacol. 2018;9:165. 29551975 10.3389/fphar.2018.00165 PMC5840524

[19] Huang C Li R Shi W. Discovery of the anti-tumor mechanism of calycosin against colorectal cancer by using system pharmacology approach. Med Sci Monit. 2019;25:5589. 31352466 10.12659/MSM.918250 PMC6683728

[20] Zhao H Shan Y Ma Z. A network pharmacology approach to explore active compounds and pharmacological mechanisms of epimedium for treatment of premature ovarian insufficiency. Drug Des Devel Ther. 2019;13:2997–3007. 10.2147/DDDT.S207823 PMC6710481 31692519

[21] Pettersen EF Goddard TD Huang CC. UCSF Chimera—a visualization system for exploratory research and analysis. J Comput Chem. 2004;25:1605–12. 15264254 10.1002/jcc.20084

[22] Zhang ND Han T Huang BK. Traditional Chinese medicine formulas for the treatment of osteoporosis: implication for antiosteoporotic drug discovery. J Ethnopharmacol. 2016;189:61–80. 27180315 10.1016/j.jep.2016.05.025

[23] Fatokun AA Tome M Smith RA. Protection by the flavonoids quercetin and luteolin against peroxide- or menadione-induced oxidative stress in MC3T3-E1 osteoblast cells. Nat Prod Res. 2015;29:1127–32. 25427161 10.1080/14786419.2014.980252

[24] Masuhara M Tsukahara T Tomita K. A relation between osteoclastogenesis inhibition and membrane-type estrogen receptor GPR30. Biochem Biophys Rep. 2016;8:389–94. 28955981 10.1016/j.bbrep.2016.10.013 PMC5614543

[25] Jing Z Wang C Yang Q. Luteolin attenuates glucocorticoid-induced osteoporosis by regulating ERK/Lrp-5/GSK-3β signaling pathway in vivo and in vitro. J Cell Physiol. 2019;234:4472–90. 30192012 10.1002/jcp.27252

[26] Ren H Sun R Wang J. Relationship of melatonin level, oxidative stress and inflammatory status with osteoporosis in maintenance hemodialysis of chronic renal failure. Exp Ther Med. 2018;15:5183–8. 29904403 10.3892/etm.2018.5857 PMC5996674

[27] Li J Karim MA Che H. Deletion of p16 prevents estrogen deficiency-induced osteoporosis by inhibiting oxidative stress and osteocyte senescence. Am J Transl Res. 2020;12:672–83. 32194914 PMC7061825

[28] Baek KH Oh KW Lee WY. Association of oxidative stress with postmenopausal osteoporosis and the effects of hydrogen peroxide on osteoclast formation in human bone marrow cell cultures. Calcif Tissue Int. 2010;87:226–35. 20614110 10.1007/s00223-010-9393-9

[29] Chavan SN More U Mulgund S. Effect of supplementation of vitamin C and E on oxidative stress in osteoporosis. Indian J Clin Biochem. 2007;22:101–5. 23105693 10.1007/BF02913324 PMC3453795

[30] Khosla S Farr JN Tchkonia T. The role of cellular senescence in ageing and endocrine disease. Nat Rev Endocrinol. 2020;16:263–75. 32161396 10.1038/s41574-020-0335-y PMC7227781

[31] Farr JN Fraser DG Wang H. Identification of Senescent Cells in the Bone Microenvironment. J Bone Miner Res. 2016;31:1920–9. 27341653 10.1002/jbmr.2892 PMC5289710

[32] Genin EC Caron N Vandenbosch R. Concise review: forkhead pathway in the control of adult neurogenesis. Stem Cells. 2014;32:1398–407. 24510844 10.1002/stem.1673

[33] Webb AE Brunet A. FOXO transcription factors: key regulators of cellular quality control. Trends Biochem Sci. 2014;39:159–69. 24630600 10.1016/j.tibs.2014.02.003 PMC4021867

[34] Jensen KS Binderup T Jensen KT. FoxO3A promotes metabolic adaptation to hypoxia by antagonizing Myc function. EMBO J. 2011;30:4554–70. 21915097 10.1038/emboj.2011.323 PMC3243591

[35] Bartell SM Kim HN Ambrogini E. FoxO proteins restrain osteoclastogenesis and bone resorption by attenuating H2O2 accumulation. Nat Commun. 2014;5:3773. 24781012 10.1038/ncomms4773 PMC4015330

[36] Ghosh-Choudhury N Abboud SL Nishimura R. Requirement of BMP-2-induced phosphatidylinositol 3-kinase and Akt serine/threonine kinase in osteoblast differentiation and Smad-dependent BMP-2 gene transcription. J Biol Chem. 2002;277:33361–8. 12084724 10.1074/jbc.M205053200

[37] Xi JC Zang HY Guo LX. The PI3K/AKT cell signaling pathway is involved in regulation of osteoporosis. J Recept Signal Transduct Res. 2015;35:640–5. 26390889 10.3109/10799893.2015.1041647

[38] Li H Malhotra S Kumar A. Nuclear factor-kappa B signaling in skeletal muscle atrophy. J Mol Med (Berl). 2008;86:1113–26. 18574572 10.1007/s00109-008-0373-8 PMC2597184

[39] Zhu S Wei W Liu Z. Tanshinone-IIA attenuates the deleterious effects of oxidative stress in osteoporosis through the NF-κB signaling pathway. Mol Med Rep. 2018;17:6969–76. 29568934 10.3892/mmr.2018.8741 PMC5928650

[40] Huang J Hsu YH Mo C. METTL21C is a potential pleiotropic gene for osteoporosis and sarcopenia acting through the modulation of the NF-κB signaling pathway. J Bone Miner Res. 2014;29:1531–40. 24677265 10.1002/jbmr.2200 PMC4074268

